# Optogenetic cortical spreading depolarization induces headache-related behaviour and neuroinflammatory responses some prolonged in familial hemiplegic migraine type 1 mice

**DOI:** 10.1186/s10194-023-01628-8

**Published:** 2023-07-26

**Authors:** Anisa Dehghani, Maarten Schenke, Sandra H. van Heiningen, Hulya Karatas, Else A. Tolner, Arn M. J. M. van den Maagdenberg

**Affiliations:** 1 Department of Human Genetics, Leiden University Medical Center, Leiden, RC 2300 The Netherlands; 2 Department of Anesthesia and Critical Care and Pain Medicine, Harvard Medical School, Beth Israel Deaconess Medical Center, Boston, MA USA; 3 Institute of Neurological Sciences and Psychiatry, Hacettepe University, Ankara, Turkey; 4 Department of Neurology, Leiden University Medical Center, Leiden, RC 2300 The Netherlands

**Keywords:** Cortical spreading depolarization, Neuroinflammation; pannexin-1, HMGB1, Optogenetics, Headache behaviour

## Abstract

**Background:**

Cortical spreading depolarization (CSD), the neurophysiological correlate of the migraine aura, can activate trigeminal pain pathways, but the neurobiological mechanisms and behavioural consequences remain unclear. Here we investigated effects of optogenetically-induced CSDs on headache-related behaviour and neuroinflammatory responses in transgenic mice carrying a familial hemiplegic migraine type 1 (FHM1) mutation.

**Methods:**

CSD events (3 in total) were evoked in a minimally invasive manner by optogenetic stimulation through the intact skull in freely behaving wildtype (WT) and FHM1 mutant mice. Related behaviours were analysed using mouse grimace scale (MGS) scoring, head grooming, and nest building behaviour. Neuroinflammatory changes were investigated by assessing HMGB1 release with immunohistochemistry and by pre-treating mice with a selective Pannexin-1 channel inhibitor.

**Results:**

In both WT and FHM1 mutant mice, CSDs induced headache-related behaviour, as evidenced by increased MGS scores and the occurrence of oculotemporal strokes, at 30 min. Mice of both genotypes also showed decreased nest building behaviour after CSD. Whereas in WT mice MGS scores had normalized at 24 h after CSD, in FHM1 mutant mice scores were normalized only at 48 h. Of note, oculotemporal stroke behaviour already normalized 5 h after CSD, whereas nest building behaviour remained impaired at 72 h; no genotype differences were observed for either readout. Nuclear HMGB1 release in the cortex of FHM1 mutant mice, at 30 min after CSD, was increased bilaterally in both WT and FHM1 mutant mice, albeit that contralateral release was more pronounced in the mutant mice. Only in FHM1 mutant mice, contralateral release remained higher at 24 h after CSD, but at 48 h had returned to abnormal, elevated, baseline values, when compared to WT mice. Blocking Panx1 channels by TAT-Panx_308_ inhibited CSD-induced headache related behaviour and HMGB1 release.

**Conclusions:**

CSDs, induced in a minimally invasive manner by optogenetics, investigated in freely behaving mice, cause various migraine relevant behavioural and neuroinflammatory phenotypes that are more pronounced and longer-lasting in FHM1 mutant compared to WT mice. Prevention of CSD-related neuroinflammatory changes may have therapeutic potential in the treatment of migraine.

## Introduction

Migraine is a common brain disorder characterized by attacks of severe headache and other neurological symptoms [1]. In one-third of patients, an aura precedes the headache; hence the subtypes migraine with and migraine without aura. In experimental animals, cortical spreading depolarization (CSD), the neurophysiological correlate of the aura, was shown to activate trigeminal pain pathways in animals [2–4], but compelling evidence what underlies the clinical features from human studies is largely lacking and difficult to obtain. Hence, there is a clear need to further improve animal experimentation and increase their translation value.

Until now, studies to investigate consequences of CSD in animals have almost always used invasive methods that involve craniotomy or pinprick, and/or investigated mice under anesthesia to trigger events. In rats, CSD induced by invasive pinprick, KCl application to the dura or electrical stimulation was found to activate neurons in the trigeminovascular system [5–7] and shown to cause putative pain-related behaviour, as indicated by, for instance, freezing behaviour, hypomobility, head shaking and/or wet-dog shakes [8–11]. Experimentally induced CSD by invasive cortical pinprick or topical KCl application on the dura in mice was shown to also activate neuroinflammatory responses [12–15]. Karatas and co-workers [12] revealed that a cascade of events after CSD, starting with the opening of neuronal Pannexin-1 (Panx1) channels, followed by activation of caspase-1 triggering neuronal release of high mobility group box 1 (HMGB1) protein - the innate ‘alarmin’ molecule - and secretion of inflammatory molecules, led to meningeal activation and ultimately a pain mimic, as assessed by the mouse grimace scale (MGS) score. The MGS score compiles mouse facial features from photo or video collages of ‘no-pain’ (baseline) and ‘pain’ conditions [16] and has repeatedly been shown a reliable preclinical pain assessment tool in rodents [17, 18]. CSD was also shown to induce proinflammatory gene expression changes (including changes in Panx1 expression) in the cortex of rodents [14, 19–21], with some changes relying on Panx1 activation [14]. The invasive methodology used for induction of CSD events in the studies described above, however, result in compromised brain tissue that already can cause skull or meningeal inflammation leading to meningeal nociceptor activation [22]. Thus, both the magnitude and time course of neuroinflammatory responses after CSD, as well as possible activation of headache mechanisms and behavioral pain mimics, are likely confounded.

A recent advancement to overcome such caveats is the development of a minimally invasive method to induce CSD events using optogenetics in mice that express blue light sensitive channelrhodopsin-2 in cortical neurons [23]. Only a few studies have investigated consequences of optogenically induced CSD, be it on neuroinflammation [20] or behaviour [24–26]. Until now, behavioural consequences were studied only shortly after (a few min to 30 min) optogenetic-induced CSD, for instance in the context of sleep-wake states [24], and typical behavioural features – abnormal locomotion, increased freezing, head shaking, prolonged facial grooming –, earlier reported with KCl-induced CSD, were also observed [24, 26]. In only one study MGS scores, as a pain mimic, were investigated and found to be increased two days after repeated (two weeks, every other day) – but not single – induction of CSD, with the caveat that mice had coverslips placed over large craniotomies and were kept under anaesthesia every time CSDs were induced [27].

To overcome the confounders from previous studies, we here combined, in freely behaving mice, optogenetic induction of CSDs through the intact skull with an assessment of headache-related behaviour for a prolonged period of time. Unique to this study is that we also assessed whether neuroinflammatory changes were present.

As CSD events were induced after one week of recovery from surgery (necessary to place the optic fibre and recording electrodes) confounding effects of surgery and anaesthesia are prevented. After CSD induction and behavioural assessment, brains were harvested to also assess neuroinflammatory changes. To further increase translational value, we here investigated familial hemiplegic migraine type 1 (FHM1) mutant mice that carry the human S218L missense mutation in the *Cacna1a* gene encoding the α_1A_ subunit of voltage-gated Ca_V_2.1 calcium channels [28] to assess whether outcomes were different from those in WT mice. Inducing CSDs with invasive methods is easier in FHM1 S218L mutant mice [28–30], due to increased cortical excitatory neuronal activity [31]. Moreover, the mutant mice also show increased cortical neuroinflammation at baseline [32] and after CSD [15, 33]. Thus, FHM1 mutant mice allow the study of neuroinflammation and pain behaviour in the context of an intrinsically hyperexcitable brain with an increased susceptibility to CSD-related neuroinflammatory changes [34].

To compare the impact of CSD on headache features in freely behaving WT and FHM1 mutant mice, we used MGS scores, oculotemporal strokes (head grooming), and nest building performance, as they are known measures of (head) pain [16–18, 35–39]. All behaviours were analysed at baseline and at different time points following optogenetic induction of CSD. To identify in which way neuroinflammatory responses may underlie observed CSD-related behavioural changes, we investigated cortical nuclear release of neuroinflammatory molecule HMGB1, also at various time points after CSD. Given the role of Panx1 channels in CSD-induced changes in WT mice [12], we also tested whether pre-treatment with a Panx1 inhibitor could prevent CSD-induced behavioural and neuroinflammatory changes. By circumventing confounders of invasive surgery and anaesthesia when inducing CSDs and investigating their consequences on behaviour and neuroinflammation in mice with a hemiplegic migraine mutation, we mimic migraine pathophysiology closer to the human condition than what has been tried before.

## Materials and methods

### Animals

Wildtype (WT) and transgenic heterozygous *Cacna1a* FHM1 S218L knock-in (KI) mice (“FHM1 mutant mice”) of 3 – 6 months (in exceptional cases mice were between 2 and 3 or between 6 and 9 months due to logistic challenges) were used for experimentation. All experimental groups included male and female mice. The mutant mice were generated by introducing the human pathogenic FHM1 S218L missense mutation in the orthologous mouse *Cacna1a* gene using a gene targeting approach [28]. Mice of the mutant strain were crossbred with transgenic Thy1-ChR2-YFP mice (strain 7612 – B6.Cg-Tg (Thy1-COP4/EYFP)18Gfng/J; Thy1/ChR2-YFP; the Jackson Laboratory, Bar Harbor, ME, USA) to obtain expression of blue light sensitive channelrhodopsin-2 in cortical neurons [40]. Mice were housed under a 12-h light/dark cycle (lights on from 6 a.m. to 6 p.m.) under standard housing conditions with food and water *ad libitum*. All experiments were carried out during the light period between 10 a.m. and 3 p.m. Of the mice used for MGS scoring, a subset was also used for oculotemporal stroke and nest building behaviour analysis; so the same mice were used for the indicated time points (NB: for the 5-h time point for MGS scoring and oculotemporal stroke analysis and for the 72-h time point for MGS scoring only few mice were investigated). A different set of mice was used for the HMGB1 experiments (as this involved sacrificing mice at specific time points) that all had a confirmed increased MGS score at 30 min (data not shown). In addition, sham MGS and sham HMGB1 experiments were performed in two separate sets of mice. Finally, for the TAT-Panx experiments a separate group of mice was used that underwent MGS scoring and oculotemporal stroke behaviour analysis at 30 min after CSD, after which they were sacrificed to assess HMGB1 release. All experimental procedures were carried out in accordance with recommendations of the European Communities Council Directive (2010/63/EU), were approved by the the local ethical committee of Leiden University and the Dutch national ethical committee, in accordance with recommendations of the European Communities Council Directive (2010/63/EU), and were carried out in accordance with ARRIVE guidelines. All efforts were made to minimize animal suffering.

### Optogenetic induction of cortical spreading depolarization in freely behaving mice

#### Surgery

**Fig. 1:**
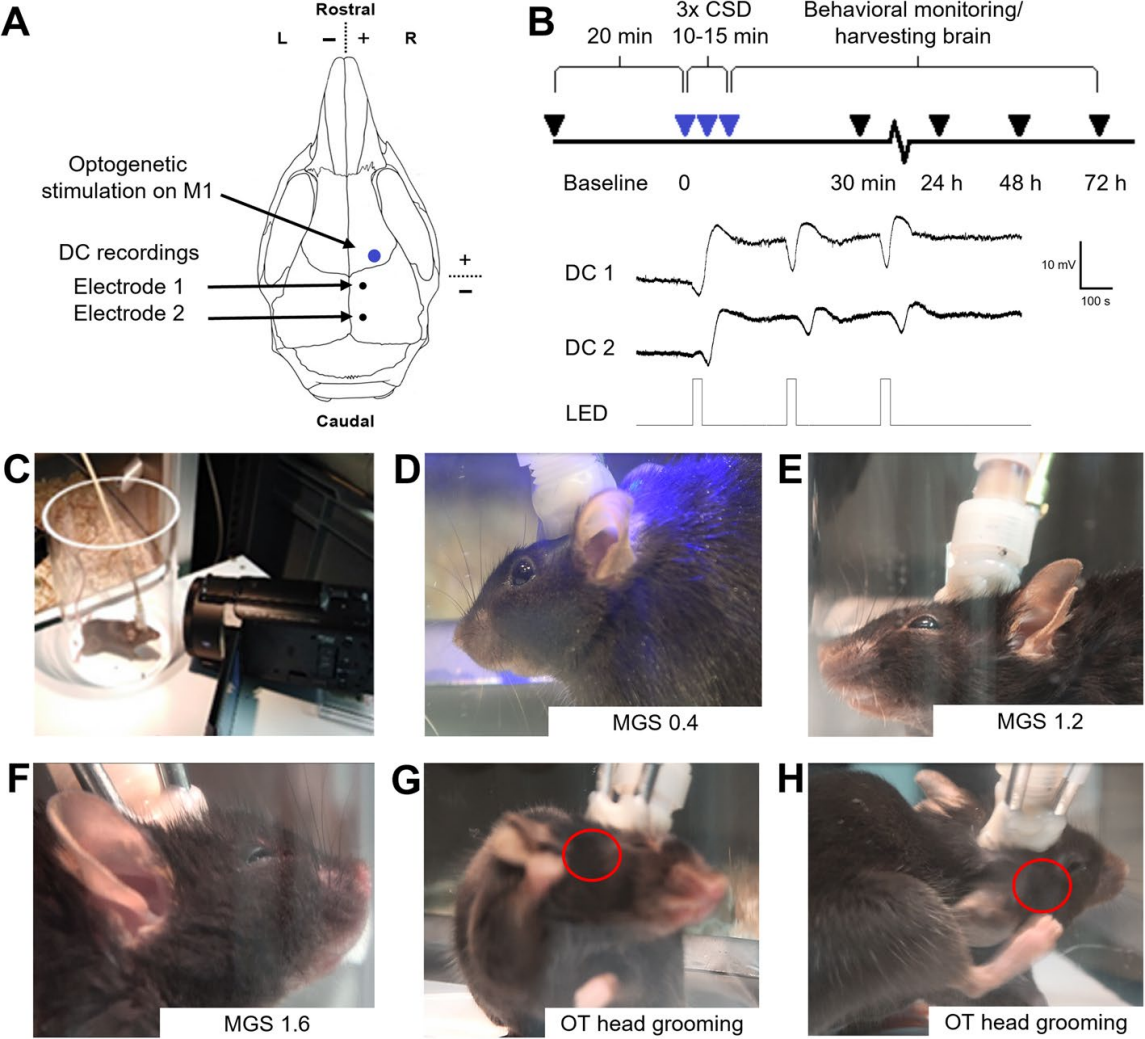
Design of optogenetic CSD induction and headache-related behavioural assessments. **A** CSDs were induced in the primary motor cortex (M1) in a minimally invasive manner by optogenetic stimulation through the intact skull. Two silver ball-tip electrodes were connected to the skull overlaying the visual and parietal cortex for direct current (DC) recording of the CSD-related DC shifts. **B** After 20-min baseline recording, three CSDs were induced optogenetically within a time window of 10-15 min. Behavioral monitoring was performed before CSD at baseline, and after CSD, i.e. at 30 min, 24 h, 48 h, and 72 h after the 3^rd^ CSD. Brains were harvested from naïve mice and mice that underwent CSD (at the four time points mentioned). **C**-**F** Mouse grimace scale (MGS) scores were monitored and recorded with examples shown, that is **C** 20 min before CSD induction; **D** at the time of CSD induction (example shown of a video-still with a MGS score of 0.4); **E** at 30 min after the 3^rd^ CSD (example shown with a MGS score of 1.2); and **F** at 72 h after the 3^rd^ CSD (example shown with a MGS score of 1.6). **G**, **H** Example video-stills of oculotemporal (OT) (head grooming) strokes in a mouse at 30 min after the 3^rd^ CSD

Mice underwent minimally invasive surgery using isoflurane anaesthesia (4 – 5% induction, 1.7 – 2% maintenance) in oxygen-enriched air, with body temperature maintained at ~37°C by a rectal probe and homeothermic blanket control unit. Small superficial indentations were drilled in the skull bone, without penetrating it, over the somatosensory (S1) and visual cortex (V1), in which silver (Ag) ball tip ~100-µm diameter (AG5493; Advent Research Materials, Witney, UK) electrodes were fixed to the skull using graphite wire glue (Anders Products, Melrose, MA, USA) at the following coordinates (mm to bregma): -1.0 posterior/1.0 lateral (right S1); -3.0 posterior/1.0 lateral (right V1). Two additional Ag ball tip electrodes on the skull above the cerebellum served as reference and ground. A fibre optic cannula (400 µm; CFM14L02; Thorlabs, Newton, NJ, USA) for photo-stimulation was placed on the skull bone over right M1 cortex 1.5 mm anterior and 1.8 mm lateral to bregma (Fig. 1 A). An additional fibre optic cannula was placed above the right V1 cortex as backup. Electrodes were connected to a pedestal (Plastics-One, Roanoke, VA, USA) and attached to the skull together with the optic fibres using UV light-activated bonding primer and dental cement (Kerr Optibond/Premise flowable; DiaDent Europe, Almere, The Netherlands). Carprofen (5 mg/kg, s.c.) was administered for post-operative pain relief.

#### Recording and induction of CSD

After a 7-day recovery from surgery period, animals were placed in a glass jar (10 cm width, 15 cm height) that allowed free movements (i.e. with no restriction of full body movements, such as rearing and grooming). The jar was placed in front of the CSD induction/recording cage and connected to custom-built recording hardware through a counterbalanced, low-torque electrical commutator. The M1 optic fibre cannula was connected via a flexible optic fibre to a 460-nm (blue) LED source (UHP-T-LED-460; Prizmatix, Givat-Shmuel, Israel). Electrophysiological signals were pre-amplified 3X and fed into separate amplifiers for direct-current (DC) potential (500 Hz low-pass filter, 10X gain, relative to reference) and local field potential (LFP; 0.05-500 Hz band-pass filter, 800X gain, relative to reference). Signals were digitized (Power 1401 with Spike2 software; CED, Cambridge, UK) at sampling rates of 1000 Hz for DC-potential and 5000 Hz for LFP signals. After a 10-min habituation to the set-up and verification of stable DC-potential signals, three consecutive CSDs, separated by 5 min, were induced at the M1 location by optogenetic stimulation [41] using a suprathreshold pulse of 460 nm blue light of 4-mW intensity and 30-s duration. Three CSDs were induced, instead of one, mainly based on the finding by Harriot et al*.* [27] that a single CSD would not enhance MGS scores in mice. In cases where no CSD could be induced by a given photostimulation, an additional stimulation at higher intensity was given to ensure three successful CSD inductions within a 15-min period. In two FHM1 mutant mice, one photostimulation evoked a CSD consisting of two further waves, in which case no further stimulation was given. CSD events were identified by their characteristic DC-deflection and spread between the two recording electrodes (Fig. 1 B). The sham stimulation group had a non-connected (‘loose’) optic fibre, whereby three consecutive stimulations were given at the M1 location with 4-mW blue light at 30-s duration, that did not provoke CSD.

### Assessment of pain-related behaviour

#### Monitoring MGS, head grooming, and nest building behaviour

Video recordings were used to obtain behavioral pain readouts (MGS, head grooming (oculotemporal strokes), and nest building performance) before, during and after CSD (Fig. 1 B-H). For MGS and head grooming, during and after optogenetic CSD induction behaviour was recorded using a full HD camera with 1920 x 1080 resolution (HDRCX625B; Sony, Tokyo, Japan**) (**50 frames/s) placed in front of the glass jar (Fig. 1 C). MGS and head grooming were also scored live in each experimental session. Starts of live sessions were 20 min before the start of CSD induction (baseline), 30 min after the first CSD and 5, 24, 48, and 72 h after CSD induction. For *post hoc* analyses, MGS and head grooming were analysed from video for the last 10 min before the first photostimulation (baseline) and for the 30- to 40-min window following the first CSD (i.e. the 20- to 30-min window following the last CSD). To rule out that the tower (containing the ball tip electrodes and optogenetic fibre cannulas) on the head of the mouse may confound assessment of pain-related behaviour, mice with and without such tower had been tested in the glass jar during pilot experiments, which revealed that all MGS features could be reliably assessed in mice with a head tower (data not shown). The head pain monitoring set up was cleaned with ethanol in between sessions with different animals, but not when it concerned different sessions of the same animal. Nest building performance was assessed in the home cage of the mouse using pressed cotton squares placed in the home cage of the mouse [36, 42]. Nest building performance was monitored and scored live on days before and 24 and 48 h after CSD induction.

#### MGS analysis

The MGS method independently scores five facial action units (FAUs; ([16, 17] on a 3-point scale for their presence and intensity, as follows: a value of 0 (not present), 1 (moderately visible) or 2 (severe). The five FAUs were: (1) orbital tightening, (2) ear position, (3) nose bulge, (4) cheek bulge, and (5) whisker position. An averaged MGS score (by averaging the 5 FAUs) was obtained for each mouse for each time point. For all sessions, live scoring was also used for baseline and the 30-min time point after CSD; for a subset of the 24-, 48- and 72-h data MGS were assessed both live and *post hoc*. For *live* s*coring* of MGS, the observer looked real-time at the mouse for 1 min and then awarded a score of 0, 1 or 2 for each of the 5 FAUs. If the mouse was grooming, sleeping, or sniffing, the score was recorded following cessation of this behaviour [16]. For *post hoc* scoring of MGS*,* videos taken during the experiments were analysed *post hoc,* for which initially two approaches were tested: (1) MGS scoring of a 1-min video-segment in which all 5 FAUs are visible and (2) MGS scoring of one frame (i.e. a still from the video at one time point) in which all five FAUs are visible. The first approach was judged superior as the 1-min video always contained all five FAUs at some time point with the required resolution and clarity for analysis, whereas the second approach did not always produce a frame in which all five FAUs were representative clearly. For the *post hoc* MGS analysis, therefore, each video was first observed as a whole, after which a 1-min time frame was chosen that was judged to best represent the MGS observed throughout the whole video. The *post hoc* observer was blinded to the genotype and experimental condition as to prevent a possible bias when scoring facial expressions.

#### Head grooming behaviour

Head grooming behaviour was scored live during all experimental time points. In addition, *post hoc* blinded analysis was performed for the baseline and 30-min time point after CSD, using the videos also used for MGS scoring. Head grooming was assessed by counting the number of oculotemporal strokes and their laterality. Strokes were defined as long forepaw or hindpaw strokes specifically directed (at their initiation) to the lateral, temporal or periorbital area of the head. Stroke laterality was assessed from the number of strokes directed to either the right, the left, or both sides (bilateral grooming), whereby the ratio of left, right and bilateral strokes was calculated. The point of initial contact of the hind or forelimb determines the facial region to which a stroke was directed. The initial phase of grooming restricted to the mystacial vibrissae, called ‘elliptical strokes’, was not included in the assessment [35, 43]. In addition to determining ratios, a second calculation was performed in which the number of OT strokes to the right side of the head was subtracted from the number of strokes directed to the left side of the head [37], allowing the comparison of absolute values.

#### Nest building performance

Nest building performance was monitored and scored live using a definitive 5-point nest-rating scale, whereby the condition of the tightly packed nesting material, the ‘Nestlet’, which the mice need to tear and rearrange to make a nest, is judged [36, 42] as: 1 (more than 90% of Nestlet is intact), 2 (50–90% of Nestlet is intact), 3 (less than 50% of Nestlet is intact but no identifiable nest), 4 (an identifiable but flat nest: more than 90% of the nestlet is torn), and 5 (a (near) perfect nest: more than 90% of the Nestlet is torn and the nest is a crater, with walls higher than mouse body height for more than 50% of its circumference).

#### Pharmacological intervention

Panx1 channel inhibitor TAT-Panx_308_ (5 mg/kg in saline) or its scrambled protein TAT-Panx_sc_ (5 mg/kg in saline) (both gifts from Dr. Roger J. Thompson, University of Calgary, Calgary, Alberta, Canada) [44] was applied i.p. 30 min before optogenetic induction of CSD. TAT-Panx_308_ is a peptide that mimics the Y308 phosphorylation site localized in the C-terminus of Panx1. The peptide contains a characteristic TAT sequence allowing for easy cell penetration. Exposure of cells to this peptide prevented Panx1-Y308 phosphorylation and selectively inhibited the opening of the channel [44].

### Immunohistochemistry

Brain tissues were collected from naïve (untreated) mice and mice that underwent optogenetically induced CSD (i.e. at 30 min, 24 h or 48 h following the last CSD) after transcardial perfusion via a quick, short (1-min) phosphate-buffered saline infusion followed by 4% PFA for 4 min. Such protocol avoids perfusion-related hypoxic stress on neurons and its adverse effect on HMGB1 release [45]. Tissue was post-fixed in 4% PFA overnight and cryoprotected in 30% sucrose in PBS for two days. Coronal sections were cut at a cryostat at 20-µm thickness. The primary somatosensory cortex area (S1) was used for HMGB1 analysis as it was shown before that this area shows a high amount of neuronal HMGB1 release following CSD [15]. Sections were incubated with primary rabbit polyclonal HMGB1 antibody (1:200; Abcam, Cambridge, MA, USA) for 24 h at 4°C [15]. Next, sections were incubated with secondary goat-anti-rabbit Cy3 antibody (1:200; Jackson ImmunoResearch, West Grove, PA, USA) for 90 min at room temperature. Immunolabeled sections were mounted in 1:1 glycerol:PBS medium containing 12.5 mg/mL sodium azide and 1 µL/mL Hoechst-33258 and examined under a wide-field fluorescence or laser-scanning confocal microscope with appropriate filter sets.

### Quantification of HMGB1 release

All quantifications were performed *post hoc* from microscopic images using ImageJ (version 1.52q) by an observer that was blinded to the genotype and experimental condition. HMGB1-labeled nuclei were quantified in the deep layers of the S1 cortex of the left and right hemisphere using microscopic images made at 200X magnification, as described before [15]. In WT mouse brain tissue, the majority of cortical neurons was described to be HMGB1-positive [12, 15]. The number of HMGB1-positive nuclei, as assessed by Hoechst staining, reflects the number of HMGB1 positive neurons [15]. Here, using Hoechst as nuclear marker, a Hoechst-positive/HMGB1-negative cell was counted as cell with ‘total HMGB1 release’, and a Hoechst-positive/HMGB1-positive signal was regarded as a cell with ‘no HMGB1 release’.

### Statistical analyses

The effect size was measured based on comparable data from previous studies [12, 15]. With an alpha of 0.05 and power at 0.80, the sample size suitable for this effect size is estimated at five/group. For behavioral scores, with the similar alpha and power and a standard deviation of 0.28, sample size is estimated to be six/group [27, 46]. Prior to statistical analysis, the Shapiro–Wilk and Anderson-Darling normality tests were applied, which revealed a non-normal distribution of data thus requiring nonparametric testing. For the analysis of the MGS, oculotemporal stroke, nest building, and HMGB1 data at different time points following CSD, the Kruskal-Wallis test was used, followed by *post hoc* pairwise comparisons with Mann-Whitney *U* tests. Critical *p*-values were adjusted for multiple testing by Bonferroni correction (as mentioned in the text). For two-group comparisons, a Mann-Whitney *U* test was used. A *p*-value <0.05 was considered significant. Data are expressed as mean with standard error of the mean (mean ± SEM). Statistical analysis was performed using GraphPad Prism 8 (GraphPad Software Inc, San Diego, CA, USA). Significant data are indicated by one (* = *p*-value 0.01- 0.05), two (** = *p*-value 0.001-0.01), three (*** = *p*-value <0.001) or four (****= *p*-value <0.0001) asterisks.

## Results

### Optogenetic CSD triggers more prolonged headache-related behaviour in FHM1 mutant compared to WT mice

**Fig. 2:**
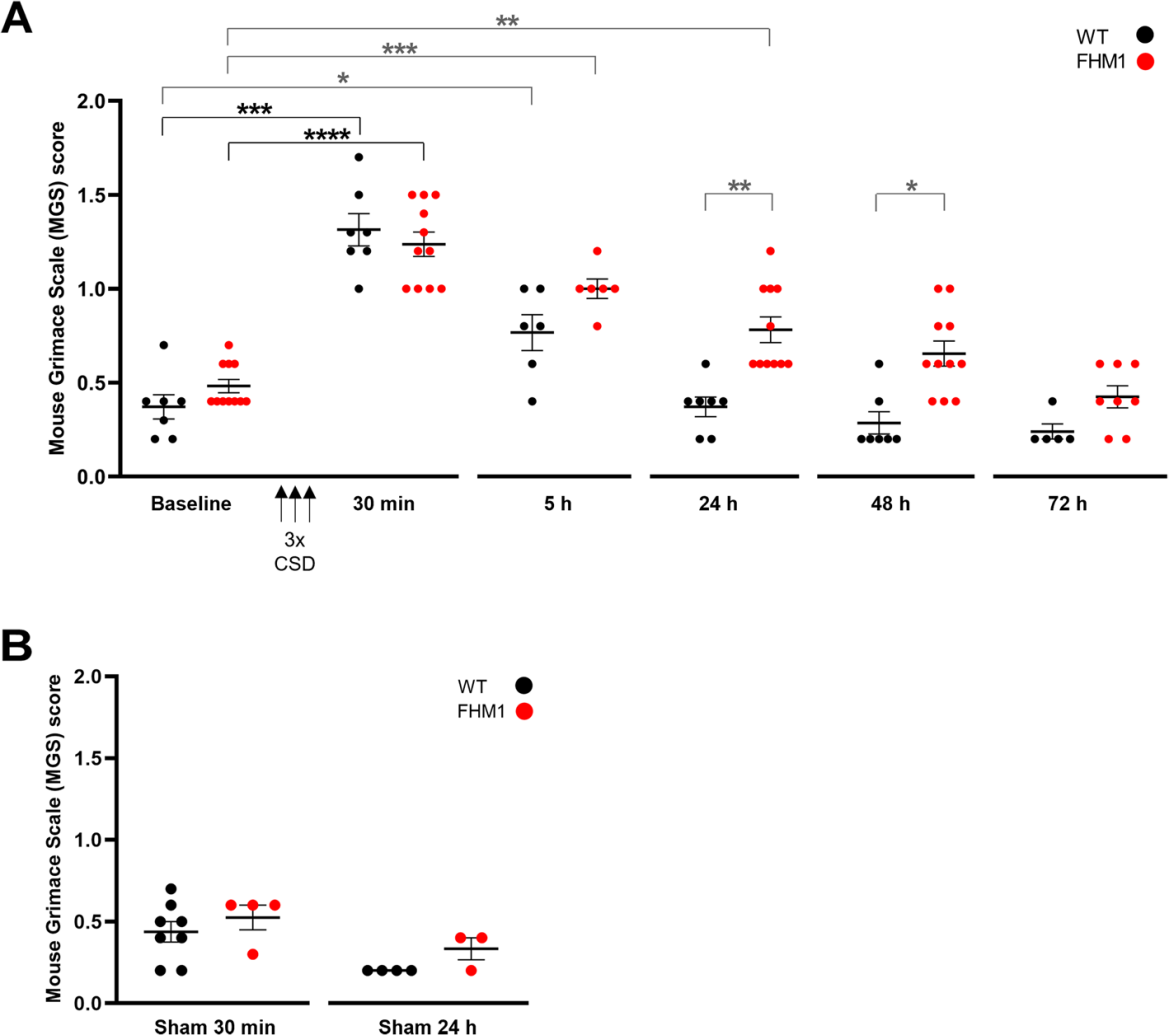
Optogenetic CSDs trigger a prolonged elevation of mouse grimace scale (MGS) scores in freely behaving FHM1 mutant mice followed over time. **A** MGS scores are increased at 30 min after three optogenetically-induced CSDs (‘3x CSD’) in both WT and FHM1 mutant mice. At 5 h after CSD, MGS scores had reduced but remained elevated compared to baseline in both genotypes. Whereas in WT mice scores had returned to baseline levels at 24 h following CSD, scores in FHM1 mutant mice had normalized only at the 48-h time point. Compared to WT mice, MGS scores were higher in FHM1 mutant mice at both 24 and 48 h following CSD. **B** In sham-treated mice, which had undergone the same surgical procedures with a head-mount including electrodes but a ‘loose’ optic fiber placement, that allowed blue light stimulation without induction of a CSD, MGS scores were not different from baseline at 30 min and 24 h after photo-stimulation in both genotypes, with no genotypic difference. * = *p* < 0.05, ** = *p* < 0.01, *** = *p* < 0.001, and **** = *p* < 0.0001. Black asterisks and brackets: uncorrected *p*-values. Grey symbols: corrected *p*-values (after Kruskall-Wallis; see Results text for number and nature of corrections)

Following optogenetic induction of three CSD events, MGS values (assessed at 30 min, 5, 24 48 and 72 h) were changed compared to baseline (*p* < 0.0001, Kruskal-Wallis test). MGS scores were increased in the 20- to 30-min window after the last CSD event (i.e. 30 – 40 minutes after the first CSD event; from here on referred to as the ‘30-min time point’) in both WT and FHM1 mutant mice (WT: *p* = 0.0006, *n* = 7; FHM1: *p* = 0.0001, *n* = 11; Fig. 2 A). Next, we investigated the return to baseline after the 30-min time point (*p*-values corrected for 4 comparisons, i.e. 5, 24, 48, and 72 h). Five h following CSD, MGS scores were still increased compared to baseline in WT (*p* = 0.030; *n* = 6) and FHM1 mutant mice (*p* = 0.0004; *n* = 6). In WT mice, MGS values normalized at 24 h (*p* > 0.99; *n* = 7; Fig. 2 A). In contrast, in FHM1 mutant mice, MGS values had not normalized to baseline at 24 h (*p* = 0.0024; *n* = 11) but did no longer differ from baseline at 48 h (*p* = 0.19; *n* = 11). MGS values at both time points were higher in FHM1 mutant compared to WT mice (24 h: *p* = 0.0010; 48 h: *p* = 0.011; corrected for 5 comparisons, i.e. 30 min, 5, 24, 48, and 72 h). As control (‘sham’) experiment, we tested the presence of a non-connected (‘loose’) optic fibre in a similar tower, in combination with blue light photostimulation on the motor cortex, which did not provoke CSD. In comparison to naïve WT/Thy1-ChR2 mice without a tower (*n* = 3), WT mice with a tower (*n* = 7) did not have different MGS scores (naïve: 0.13 ± 0.07; sham unstimulated: 0.37 ± 0.06; *p* = 0.075). For FHM1 mutant mice without a tower (*n* = 3) MGS scores were also not different than for mice with a tower (*n* = 11) (naïve: 0.40 ± 0.00; sham unstimulated: 0.48 ± 0.04; *p* = 0.51), indicating that the tower itself did not confound MGS scores for both genotypes. Moreover, at the 30-min time point following sham photostimulation, MGS scores were not different between FHM1 mutant and WT mice (*p* = 0.38; Fig. 2 B). With respect to the effect of sham stimulation compared to CSD induction on MGS over time (correction for two comparisons, i.e. 30 min and 24 h), MGS values were higher for CSD compared to sham-treated animals at the 30-min time point in both WT (*p* = 0.0006) and FHM1 mutant *(p* = 0.004) mice*.* At 24 h following CSD, MGS values were not different from those of sham-treated animals for WT mice (*p* = 0.122), while still being significantly enhanced for FHM1 mutant mice compared to sham-treated animals (*p* = 0.006). Sham-values at 24 h were not different between genotypes (*p =* 0.28) (Fig. 2 A and B).

### Optogenetic CSD causes a short-lasting increase in head grooming and prolonged impairment of nest building performance in both WT and FHM1 mutant mice

**Fig. 3:**
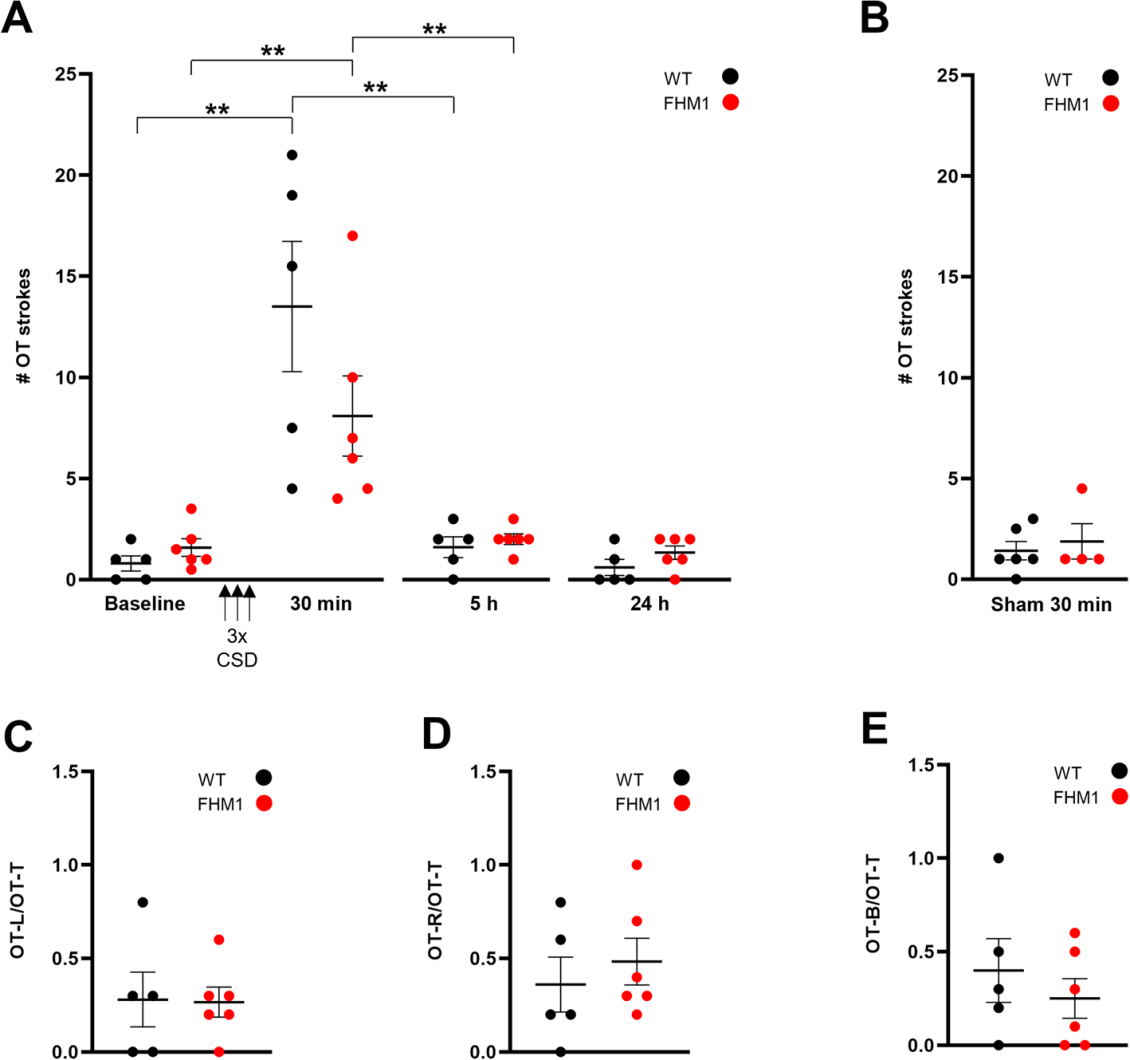
Optogenetic CSDs cause a short-lasting increase in oculotemporal (OT) (head grooming) strokes in both WT and FHM1 mutant mice. **A** In both WT and FHM1 mutant mice, the number (#) of strokes was elevated 30 min after three optogenetically-induced CSDs (‘3x CSD’) and had normalized at 5 h after CSD. **B** The number of strokes did not change with sham stimulation. **C**-**E** Strokes from **A** at 30 min showed no difference in the occurrence of left (**C**), right (**D**), or bilateral (**E**) strokes between WT and FHM1 mutant mice. OT-L/OT-T: number of left OT strokes/total; OT-R/OT-T: number of right OT strokes/total; OT-B/OT-T: number of bilateral OT strokes/total. ** *p* < 0.01

Oculotemporal strokes (i.e. specific head grooming behaviour) in rodents have been shown to reflect behaviour related to pain [35, 39, 43], including headache [37]. Following CSD, the number of oculotemporal strokes was changed (*p* = 0.0002, Kruskal-Wallis test). At 30 min after CSD, the number of strokes was increased in both WT (*p* = 0.0079; *n* = 5) and FHM1 mutant mice (*p* = 0.0022; *n* = 6) compared to baseline (Fig. 3 A). Next, we investigated the return to baseline after the 30-min time point and found that the number of strokes had normalized to baseline levels at 5 h after CSD for both WT (*p* = 0.38, *n* = 5) and FHM1 mutant (*p* = 0.27, *n* = 6) mice (without correction for multiple testing). Stroke behaviour was not different between WT and FHM1 mutant mice, neither at baseline nor at any of the time points after CSD. The ‘loose’ optic fibre sham control stimulation did not have an effect on oculotemporal stroke behaviour at the 30-min time point compared to baseline for both WT (*p* = 0.38; *n* = 6) and FHM1 mutant (*p* > 0.99; *n* = 4) mice (Fig. 3 B). The number of strokes at 30 min following stimulation was significantly increased for the CSD compared to sham groups for both WT (*p* = 0.004) and FHM1 mutant (*p* = 0.029) mice. We next assessed the laterality of strokes. At the 30-min time point, there was no difference in the laterality of CSD-induced stroke behaviour between genotypes (*p* > 0.99 left strokes; calculated as the ratio of the left and total number of strokes; *p* = 0.40 right strokes; *p* = 0.61 bilateral strokes) (Fig. 3 C-E).

**Fig. 4:**
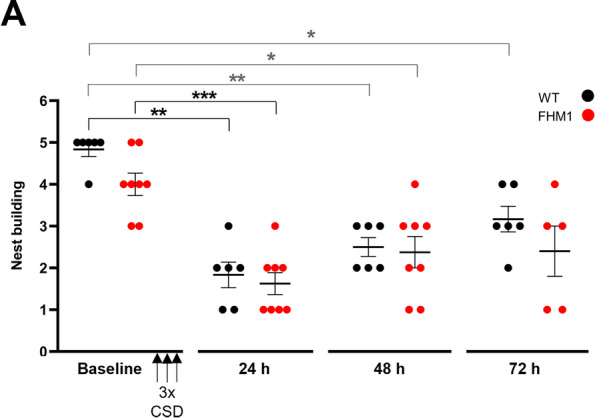
Optogenetic CSDs cause a prolonged impairment of nest building behaviour in both WT and FHM1 mutant mice. Nest building behaviour was impaired at 24 h, and had still not normalized at 48 h, after optogenetically induced CSDs in both genotypes, with no genotypic difference, and had even not returned to baseline at the 72-h time point. * *p* < 0.05, ** *p* < 0.01, *** = *p* < 0.001. Black asterisks and brackets: uncorrected *p*-values. Grey symbols: corrected *p*-values (after Kruskall-Wallis; see Results text for number and nature of corrections)

Finally, nest building performance, a more general behavioral indicator of discomfort in mice [38, 42], was also affected by CSD (*p* = 0.0002, Kruskal-Wallis test). Nest building performance after CSD was decreased in both WT (*p* = 0.0022; *n* = 6) and FHM1 mutant (*p* = 0.0005; *n* = 6) mice, at 24 h, in comparison to baseline. Next, we investigated the return to baseline after the 24-h time point (*p*-values corrected for 2 comparisons, i.e. 48 and 72 h). Nest building performance remained impaired at 48 h after CSD in both genotypes (WT: *p* = 0.0044 *n* = 6; FHM1: *p* = 0.018; *n* = 8), whereas nest building performance was similar for both genotypes (*p* = 0.063) at baseline (Fig. 4). Even at 72 h after CSD nest building performance was not fully back to baseline (WT: *p* = 0.013; FHM1: *p* = 0.071), implying that the discomfort experienced by the mice following CSD is long-lasting.

### Optogenetically-induced CSD triggers a more prolonged cortical neuroinflammatory response in FHM1 mutant compared to WT mice

**Fig. 5:**
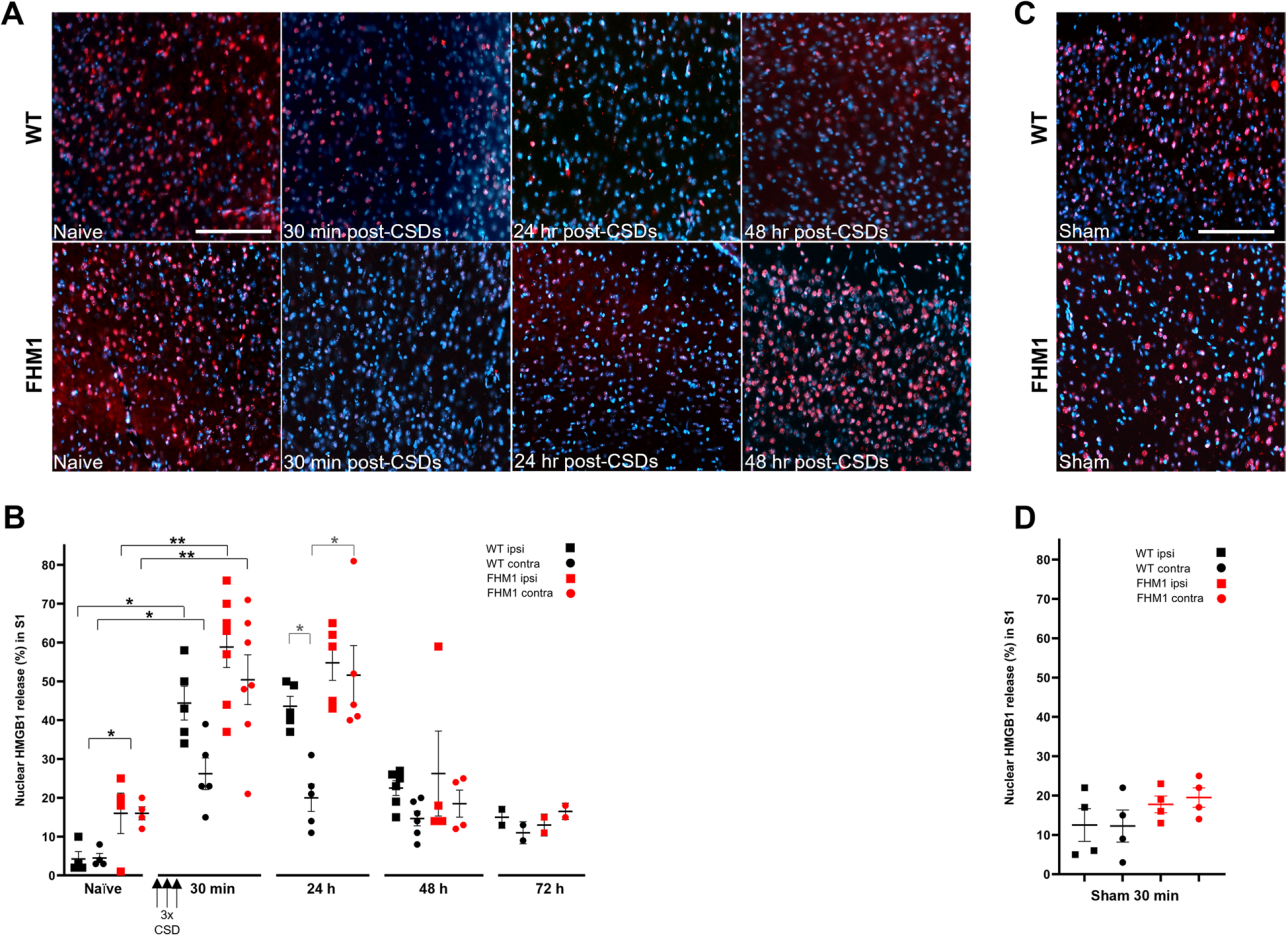
Optogenetic CSDs trigger a prolonged neuroinflammatory response in the cortex of FHM1 mutant compared to WT mice. **A** Representative photomicrographs of HMGB1 immunolabeling of the primary somatosensory cortex (ipsilateral to the side of optogenetic CSD induction) of a WT and a FHM1 mutant mouse. HMGB1 immunolabeling is shown as red fluorescence signal. In naïve (untreated) mice, HMGB1 is located in cell nuclei as indicated with co-labeling with Hoechst-33258 (blue). CSD triggers massive release of HMGB1 from neuronal nuclei to the extracellular space 30 min after three optogenetically-induced CSDs (‘3x CSD’) in both WT and FHM1 mutant mice. At 24 h, in the ipsilateral cortex, release was still pronounced in both WT and mutant mice and less so at 48 h in both groups. **B** Quantification of the % of cells displaying nuclear HMGB1 release showed that release was higher in naïve (untreated) FHM1 mutant compared to WT mice. At 30 min, both ipsi- and contralateral release were increased in both FHM1 mutant and WT mice, with ipsilateral release being higher than contralateral release in WT mice, whereas in FHM1 mutant mice release was equally high in both hemispheres. Of note, contralateral release was higher in FHM1 mutant mice than WT mice at this time point. At 24 h after CSD, HMGB1 release remained elevated compared to baseline in the ipsilateral and contralateral cortex for both WT and FHM1 mutant mice. Compared to WT mice, at 24 h, FHM1 mutant mice showed a more pronounced HMGB1 release in the contralateral cortex. At 48 h following CSD, cortical HMGB1 release in WT mice remained increased compared to baseline levels whereas release had normalized in both hemispheres for FHM1 mutant mice, but note that baseline levels in FHM1 mutant mice are elevated compared to WT. **C** Representative photomicrographs of HMGB1 immunolabeling of the primary somatosensory cortex (ipsilateral to the side of sham stimulation) of a WT and a FHM1 mutant mouse. **D** HMGB1 release in the ipsi- and contralateral cortex is not affected by sham stimulation in either genotype. * *p* < 0.05, ** *p* < 0.01. Black asterisks and brackets: uncorrected *p*-values. Grey symbols: corrected *p*-values (after Kruskall-Wallis; see Results text for number and nature of corrections). Scale bar: 250 μm (applicable to all photomicrographs)

Experimentally induced CSD (using invasive methodology) was shown to cause profound parenchymal neuroinflammatory responses in the cortex of WT mice at 30 min following CSD that involved release of HMGB1 protein (assessed as cells with nuclear release), which was most pronounced ipsilaterally [12, 15]. In naïve (i.e. untreated) mice, the number of cells with total HMGB1 release was higher in FHM1 mutant compared to WT mice (*p* = 0.0096; both *n* = 4 (both hemispheres combined)), similar to what was found before [15]. Optogenetic CSD induction resulted in a change in HMGB1 release (*p* < 0.0001, Kruskal-Wallis test). At 30 min after CSDcortical HMGB1 release was increased bilaterally in both WT (*n* = 5) and FHM1 mutant (*n* = 7) mice compared to naïve mice (WT ipsilateral and contralateral both *p* = 0.016; FHM1 ipsi- and contralateral both *p* = 0.0061; Fig. 5 A and B). Given the many possibilities (ipsi *vs* contra, WT *vs* FHM1, and various time points) it is challenging *per se* to investigate possible specific differences in paired comparisons while correcting for the various comparisons. Hence, we first focussed on the return to baseline after the 30-min time point (*p*-values for comparisons within a genotype over time corrected for 2 comparisons, i.e. 24 and 48 h; separately analyzed for ipsi- and contralateral). In addition, we investigated differences between genotypes or between ipsi- and contralateral sites within a genotype across time points (*p*-values for 3 comparisons, i.e. 30 min, 24, and 48 h). At 24 h after CSD, HMGB1 release remained elevated compared to baseline in the ipsilateral and contralateral cortex for both WT (*n* = 5) and FHM1 mutant (*n* = 5) mice (WT 24 h *vs* naïve for ipsilateral: *p* = 0.032, WT 24 h *vs* naïve for contralateral: *p* = 0.032; FHM1 24 h *vs* naïve for ipsilateral: *p* = 0.032; FHM1 24 h *vs* naïve for contralateral: *p* = 0.032). Compared to WT mice, at 24 h, FHM1 mutant mice showed a more pronounced HMGB1 release in the contralateral cortex (*p* = 0.024). Accordingly, at this time point, the increase in HMGB1 release was more pronounced ipsilaterally in WT mice (ipsi *vs* contra: *p* = 0.024), whereas no laterality was observed for FHM1 mutant mice (ipsi *vs* contra: *p* > 0.99). At 48 h following CSD, cortical HMGB1 release in WT mice remained increased compared to baseline (*n* = 5; ipsi: *p* = 0.019; contra: *p* = 0.029), whereas release had normalized in both hemispheres of FHM1 mutant mice (*n* = 5; ipsi: *p* > 0.99; contra: *p >* 0.99), but note that baseline levels in FHM1 mutant mice are elevated compared to WT. Anecdotally, at 72 h after CSD, values of both ipsi- and contralateral HMGB1 release, for both WT and FHM1 mice (*n* = 2 for both groups), were lower than 20%, which is comparable to values in naïve mice. The ‘loose’ optic fibre sham control experiments did not cause an enhancement of cortical HMGB1 release at the 30-min time point compared to naïve mice for both WT (*n* = 4; ipsi: *p* = 0.11 and contra: *p* = 0.20) and FHM1 mutant (*n* = 4; ipsi: *p* = 0.89 and contra: *p* = 0.40) mice, with no difference between the genotypes (WT *vs* FHM1 ipsi:* p* = 0.49; WT *vs* FHM1 contra:* p* = 0.23; Fig. 5 C-D). In comparison to sham stimulation, the CSD effect at 30 min largely concerned unilateral release in WT mice (ipsi: *p* = 0.0048; contra: *p* = 0.048) and bilateral HMGB1 release in FHM1 mutant mice (ipsi and contra: *p* = 0.0095).

### Inhibition of Panx1 channels prevents the acute elevation of headache-related behaviour and HMGB1 release of optogenetic CSD

**Fig. 6:**
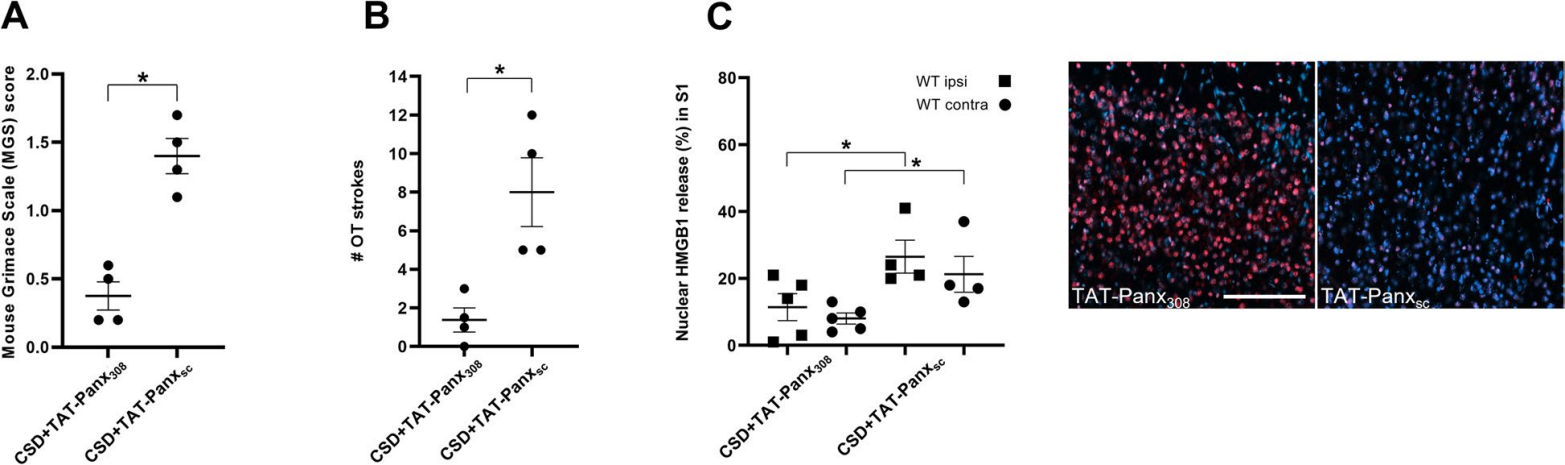
Inhibition of Panx1 channels prevents the CSD-related acute (at 30 min) elevation of MGS scores, oculotemporal (OT) (head grooming) strokes, and cortical nuclear HMGB1 release. **A** Pre-treatment of WT mice with Panx1 channel inhibitor TAT-Panx_308_ (i.p. administration 30 min prior to the start of optogenetic stimulation) prevented the elevation of MGS scores at 30 min following CSD, compared to mice pre-treated with scrambled (control) protein (TAT-Panx_sc_). **B** TAT-Panx_308_ pre-treatment prevented the increase of strokes at 30 min following CSD, compared to control TAT-Panx_sc_ administration. **C** TAT-Panx_308_ pre-treatment prevented the increase nuclear HMGB1 release in both the ipsi- and contralateral to somatosensory cortex, compared to control TAT-Panx_sc_ administration. **D** Representative photomicrographs of ipsilateral somatosensory cortex of WT mice 30 min after CSD, pretreated with TAT-Panx_308_ or TAT-Panx_sc_, stained for HMGB1 (red; nuclear Hoechst staining in blue). * *p* < 0.05. Scale bar: 250 μm

Next, we studied whether the acute effects of CSD on headache-related behaviour and cortical HMGB1 release could be prevented by blockade of neuronal Panx1 channels, as these channels open within minutes following a CSD and mediate an inflammatory cascade as part of trigeminovascular activation [12]. Only WT mice were used as we set out to investigate the mechanistic involvement of Panx1 channels *per se* on the studied parameters. To investigate effects of Panx1 channel inhibition, we used selective inhibitor, TAT-Panx_308_, which is an interfering peptide that mimics the C-terminal epitope of Panx1 including the Y308 site and blocks activation of Panx1 channels [44]. Administration of TAT-Panx_308_ prior to CSD induction in WT mice (*n* = 4), prevented the CSD-related increase in MGS scores at 30 min, yielding scores comparable to baseline. MGS score were significantly lower than the scores obtained from mice pre-treated with the ‘scrambled’ (control) protein TAT-Panx_308-Sc_ (*n* = 4; *p* = 0.029; Fig. 6 A). Similarly, pre-treatment with TAT-Panx_308_ prevented the CSD-related acute increase in OT strokes at 30 min after CSD, whereas treatment with the control TAT-Panx_sc_ did not (*p* = 0.029; Fig. 6 B). Finally, inhibition of Panx1 channels with TAT-Panx_308_ (*n* = 5) also decreased CSD-induced HMGB1 release at the 30-min time point after CSD in comparison to mice that received the scrambled protein TAT-Panx_308-Sc_ (*p* = 0.040 for the ipsilateral and *p* = 0.024 for the contralateral cortex; *n* = 4; Fig. 6 C).

## Discussion

In this study, using wildtype and transgenic hemiplegic migraine mutant mice, we showed that three CSD events induced in a minimally invasive manner by optogenetics, evoke a temporary headache-relevant (i.e. increased mouse grimace scale (MGS) scores and the presence of oculotemporal strokes) and brain neuroinflammation (i.e. HMGB1 release) phenotypes within 30 min. In FHM1 mutant mice, MGS scores normalized at 48 h, whereas in WT mice they had already returned to baseline at 24 h. At the 24 h time point, contralateral neuroinflammation was enhanced in FHM1 mutant when compared to WT mice. Neuroinflammatory responses at 48 h in FHM1 mutant mice had returned to abnormal, elevated, baseline values, when compared to WT mice. In WT mice, neuroinflammatory responses, although persisting up to 48 h, levels for both hemispheres were similar to those of FHM1 mutant mice. With respect to the oculotemporal strokes, which are characterized as long strokes specifically directed (at their start) to the lateral, temporal or periorbital area of the head, they had normalized already at 5 h after CSD and did not show laterality or a genotypic difference. Moreover, the observation that nest building behaviour had not returned to normal levels at 48 h (and even at 72 h) in either genotype indicates that reduced well-being of the mouse is long-lasting, even though headache-relevant while neuroinflammation phenotypes have already normalized. Finally, systemic administration of a selective inhibitor of Panx1 mega-channels before CSD induction, that are activated by neuronal stressors - such as CSD - and that induce a neuroinflammatory cascade [12], prevented the headache-relevant and neuroinflammatory phenotypes in WT mice suggesting that modulation of Panx1 may have therapeutic potential.

Our optogenetic approach to induce CSD events, unlike the mostly used invasive procedures, was uniquely combined with the assessment of CSD consequences in freely behaving mice over a long period of time. In other studies optogenetics has been used to induce CSD but only the behavioural consequences typically within the first half hour were investigated [24–26] and focus was on immediate behavioural responses, such as locomotion, freezing, head shaking and grooming, but not on longer lasting headache-relevant behaviour. Only the study of Harriott et al*.* [27] investigated longer lasting behaviour after CSD and also used MGS scoring, as a pain mimic, but in their procedure mice underwent repeated CSDs on multiple days with the additional caveat that mice had coverslips placed over craniotomies and mice were kept under anaesthesia when CSD was induced. This may explain the high MGS scores (typically above 0.6) in their sham controls. As the study of Harriott et al*.* [27] was not able to show raised MGS scores after a single CSD, we induced multiple (three) CSDs.

Our study is the first to induce CSD events at a particular moment and follow subsequent headache-relevant behaviour over the next few days. This and the fact that we did not use invasive procedures gives our approach particular clinical translational value, as migraines are not typically associated with injury [3]. To exclude the possibility that visual perception of the strong intensity of blue light used to induce CSDs, and not the CSD itself, had produced the behavioural responses, we performed sham experiments in which the optical fibre was loosely attached to the tower producing a blue light without triggering CSD. MGS scores were not increased in the sham-treated animals of both genotypes. Scores of the sham-treated animals were also not different from those in the respective naïve mice, proving that not the head-mount itself but the induced CSDs produced the headache-related behavior.

In our study, we focussed on several types of behaviour that had been related to (head) pain, of which MGS scoring [16, 27, 37] and the assessment of oculotemporal strokes [37] had already been used in a migraine context. New in the context of migraine is nest building performance [36], which was used to assess whether general well-being of the mouse is affected [38]. Our results show that abnormal behaviour is already present at the 30-min time point but that oculotemporal strokes normalized the fastest (already at 5 h) whereas MGS scores take longer to normalize, and nest building performance remained abnormal even after three days.

In the study, we compared consequences of CSD in WT and FHM1 mutant mice to increase translational value. The FHM1 mutant mouse model is known to exhibit neuronal hyperexcitability [28, 31, 47] associated with CSD-related [15, 33] and a basal trigeminal neuroinflammatory [48] profile. Whereas in WT mice, MGS scores remained high at 5 h but had returned to baseline at 24 h after CSD, in FHM1 mutant mice MGS scores normalized only at 48 h. Of note, for the other behavioural features, i.e. oculotemporal strokes and nest building performance, no differences between wildtype and FHM1 mutant mice were observed. Furthermore, no laterality was observed for the oculotemporal strokes, which may have been expected if this behaviour would have been specific for facial pain in the context of CSD, given that the CSD events were induced in one hemisphere. After all, the study of Chanda et al*.* [37] in FHM1 mutant mice that carried the missense R192Q [28] - and not the S218L mutation investigated in the present study - revealed evidence for stress-induced lateralized oculotemporal head grooming, which was, however, not that evident in all animals. In conclusion, in the FHM1 mutant mice the consequences of CSD, at least when it comes to the pain mimic, are longer-lasting, which seems to validate the hemiplegic migraine mouse model at the behavioural level, as migraine attacks in humans also typically last a few days [1].

Our study is also unique in that it can assess the time course of brain neuroinflammation over a prolonged period of time after minimally invasive induced CSD. Earlier, various neuroinflammatory responses were reported upon invasive (cortical pinprick or topical KCl application on the dura) CSD induction [49]. Most relevant to the present study, it was shown in WT mice that CSD induced by pinprick resulted in the opening of neuronal Panx1 channels that triggered a cascade of events that included enhanced release of the innate ‘alarmin’ molecule HMGB1 protein 30 min after CSD [12]. In a follow-up study in FHM1 mutant mice widespread neuroinflammation, i.e. bilateral HMGB1 release and NF-kB translocation in astrocytes in cortical and subcortical areas, such as thalamus, was observed up to 24 h after invasively induced CSD, whereas in WT mice such effects were only pronounced in the hemisphere in which CSD was induced [15]. Already because drilling the skull, needed to induce CSD, increases HMGB1 release [15], it is of relevance to assess neuroinflammation with our minimally invasive optogenetics paradigm and compare its development over time with that of the behavioural phenotypes.

HMGB1 release, taken here as readout of brain neuroinflammation, was increased at the 30-min time point in both genotypes. The increase in the cortex was more pronounced in the FHM1 mutant mice, where it was equally profound in both hemispheres; whereas in WT mice the increase was more prounced in the ipsilateral hemisphere, in line with previous findings [15]. Although MGS scores in WT mice are already normalized at the 24-h time point, ipsilateral HMGB1 release is still increased and was still increased at the 48-h time point. This shows that effects of CSD in WT mice can last for days. In the FHM1 mutant mice, MGS scores normalized at the 48-h time point while HMGB1 release after CSD was bilateral at all time points investigated and at 48 h had retruned to the already elevated baseline level. It is tempting to speculate that the more profound behavioural phenotype in FHM1 mutant mice correlates with widespread, i.e. bilateral, HMGB1 release. The suggestion that the intrinsic neuronal hyperexcitability phenotype of FHM1 mutant mice seems to aggravate the neuroinflammation profile, and contributes to the longer lasting pain mimics in these mice after CSD is in line with the already higher baseline level of HMGB1 release.

Finally, we could show, in WT mice, that the systemic administration prior to CSD induction of an inhibitor of Panx1 mega-channels, which are activated by neuronal stressors, such as CSD and induce a neuroinflammatory cascade [12], prevented not only the increased MGS scores and oculotemporal head grooming strokes behavioural phenotypes but also the enhanced neuroinflammatory HMGB1 release phenotype suggests that modulation of Panx1 may be a promising avenue to treat migraine.

## Conclusions

Our minimally invasive optogenetic CSD induction approach eliminates several confounding factors (craniotomy, anesthesia) present in most of the previous studies. Using freely behaving WT and transgenic hemiplegic migraine mutant mice, we could show, for the first time, headache-relevant behavioral and neuroinflammatory features for a prolonged time period after induction of CSD. Headache-relevant behaviour and cortical HMGB1 release were present at 30 min after CSD in both genotypes but lasted longer and/or were more pronounced in FHM1 mutant mice. Both were prevented by an inhibitor of Panx1 channels in WT mice. Our approach has strong translational potential by allowing the investigation of the consequences of (repeated) CSD as well as the screening of promising migraine therapeutics.

## Data Availability

The datasets from the current study are available from corresponding author A.M.J.M.v.d.M. upon reasonable request.

## Abbreviations

CSD: Cortical spreading depolarization
DC: Direct current
FAU: Facial action units
FHM1: Familial hemiplegic migraine type 1
fps: Frames per second
HMGB1: High mobility group box 1
i.p.: Intraperitoneal
MGS: Mouse grimace scale
Panx1: Pannexin-1
SD: Spreading depolarization
